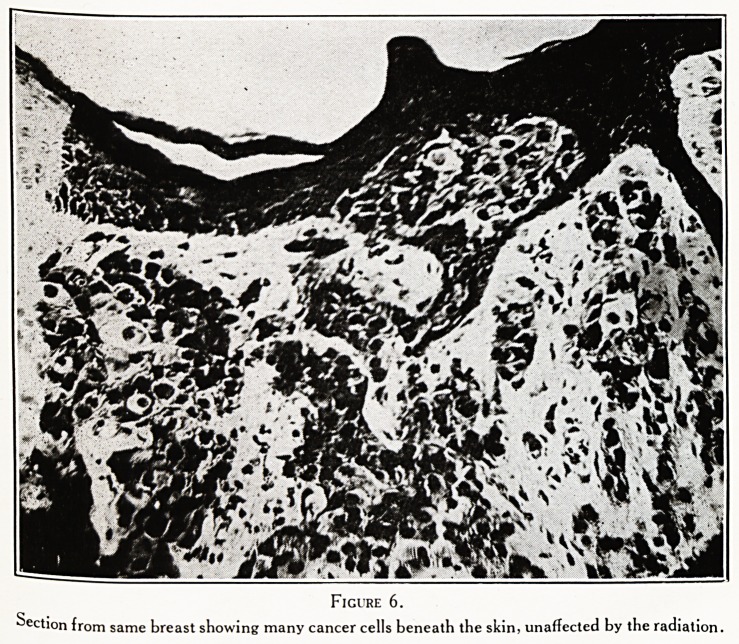# Cancer of the Breast
*Read at a Meeting of the Bristol Medico-Chirurgical Society on February 13th, 1924.


**Published:** 1924-04

**Authors:** A. Rendle Short

**Affiliations:** Surgeon to the Bristol Royal Infirmary


### CANCER OF THE BREAST.*

BY

A. Rendle Short, M.D., B.S., B.Sc., F.R.C.S.,
*Surgeon to the Bristol Royal Infirmary.*

---

Year after year the Registrar-General's commentary on the mortality statistics for England and Wales contains the melancholy note, on the death rate from cancer, that this is the highest yet recorded. All forms of cancer are increasing. In this country the increase in the death rate from malignant disease is about 2 per cent. per year ; that is to say, it rose from 800 deaths per million living to 1,000 in twelve years. In 1920 there were 43,687 deaths from all forms of cancer. As the wonderful statistics collected by Hoffman[1] show, this cannot be accounted for by the rise in the average age at death, nor by improvements in diagnosis.

Cancer of the female breast is no exception to the general tendency. In 1920 there were 4,488 deaths from this cause in women ; that is, 19 per cent. of cancer deaths in the female sex are due to cancer of the breast. Of women over 35 about 7.5 per cent. will get cancer, and in 1.5 per cent. it will affect the breast. It is not necessary to say more to show that the subject is one of great importance both to the profession and the public.

**How does Cancer of the Breast arise ?**—It is not easy to establish an exact standard as to what constitutes a normal human mammary gland in histological section. Breasts which we have no reason to suspect of disease show a good deal of variation. But in the main, in young women with healthy breasts, both the secreting acini and the ducts show

* Read at a Meeting of the Bristol Medico-Chirurgical Society on February 13th, 1924.

FIGURE 1.

Shows normal ducts, lined by a single layer of epithelium, in the upper half of the section, and ducts showing proemial changes in the lower half. At the bottom left hand corner is the focus of cancer. (Low power.)

FIGURE 2.

(Same section, high power.) Ducts showing hyperplasia of epithelium, i.e. " proemial breast.

a single lining layer of epithelium, with a well-marked basement membrane. Even the virgin mammary gland has a certain scanty secretion from the acini, which is reabsorbed in the ducts. The larger ducts at the nipple are plugged with epithelial debris.

Very commonly one finds a few ducts showing some overgrowth of the flattened cubical epithelium. This may become two or three layers thick, and so more or less completely block the lumen; or stalked outgrowths, like seaweed in a pool, may distend the duct. Naturally, if a large duct is blocked, the secretion, which very likely under these circumstances is excessive and perhaps also badly absorbed, distends the smaller channels which drain into the duct, and they become tortuous and form cysts, with, or more often without, a stalked papillomatous growth inside them. Under certain circumstances the hyperplasia or overgrowth of the duct epithelium spreads widely along the ducts; under other circumstances it bursts through the basement membrane and runs riot in the tissues outside, especially taking advantage of the lines of least resistance presented by the lymphatic vessels. Thus it has become a cancer. The fibrous-tissue reaction of the mammary gland may be normal, in which case we get a scirrhus; or excessive, presenting us with the variety called atrophic scirrhus; or the cells may overwhelm the fibrous-tissue reaction and produce the encephaloid type of cancer.

These changes are well shown in the section figured 1. One sees normal ducts and acini; ducts with some hyperplasia of the lining epithelium; ducts packed with cells that look like, and probably are, cancer cells; and a patch where the cancer cells have definitely raided the tissue after forcing a way through the basement membrane. This slide, then, shows a very early carcinoma of the breast, supervening on the chronic changes we have just described.

Modern pathologists such as Sir Lenthal Cheatle[2] in this country and Ewing[3] in America therefore describe two separate conditions as occurring in the breast, which in times past would both have been included under the title of chronic interstitial mastitis. One affects the epithelium of the ducts, and it may be the secreting acini also; to this type Cheatle gives the name of the "proemial breast," *i.e.* he regards it as a definitely pre-cancerous condition. In the other type, not so common, there is a small round-celled infiltration of the gland around the acini and ducts. This is a genuine mastitis. Most cancers of the breast, not all, therefore arise in the epithelium of the ducts, not of the acini. The disease associated with the bloody discharge from the nipple, which has of long time been known as a duct-cancer, is only a somewhat uncommon special case of cancer arising in a duct.

Thus it becomes important to us to discover why the proemial breast occurs, and how it may be recognised and treated.

The causation is obscure. According to Cheatle, an irritant gets in through the nipple ducts. In the more extreme cases of chronic intestinal stasis the breasts are usually full of the little shotty nodules which characterise the proemial breast, and Sir Arbuthnot Lane thinks an intestinal toxin is responsible. Women and girls with delayed menstruation and other signs of insufficient ovarian internal secretion may frequently show the same.

The proemial breast is seen in women from 25 to 45. In the earlier stages it may be very painful, especially at the menstrual periods. Some reddish or definitely bloody discharge can be pressed out from the nipple, being derived from the little seaweed-like growths of epithelium inside some of the ducts; this sign, however, is often absent. The breast feels "nobbly"; that is to say, there may be nothing one can definitely call a lump, but it is firm and irregular, feeling as if one had thrown a thick cloth over a pile of small shot, and was feeling the shot through the cloth. There may, of course, be little hard lumps also, probably cysts. Often a particular quadrant of the breast is alone affected. Enlarged glands may be felt in the axilla; this sign by no means proves the presence of cancer.

The treatment of this very common condition is not at all satisfactory. Excision of every proemial breast one sees is too drastic, but milder measures are not particularly effectual. It helps to support the gland with a bandage. One may prescribe Elixir Polyglandin or some other endocrine preparation, on the theory that the internal secretions, and notably that of the ovary, are at fault. This seems to do good in some cases. If there is evidence of intestinal stasis Liq. Paraffin is indicated. I have been accustomed to prescribe a blistering plaster for the painful types of this ailment.

Recently X-rays have been recommended.

In patients with this condition over 40 years of age a very close watch must be kept, and at frequent intervals, for the possible development of a definite lump such as can be felt with the flat hand or finger tips. Striking a rough average, out of 20 patients in whom this has occurred, 11 will have developed a cancer, 8 a cyst, and 1 a firm mass composed mostly of fibrous tissue, which sometimes contains in the centre a small collection of pus or liquefied epithelial debris. When a proemial breast shows a definite lump in it the whole breast tissue ought to be removed, and not only the lump. We shall return to this later.

**Ætiology of Cancer of the Breast.**—In my experience, both of hospital cases and private practice, an unduly large proportion of women suffering from mammary carcinoma are sterile; either they are spinsters, or being married they have never borne children.

In 1913 the Registrar-General analysed the figures for women dying of this disease, and corrected them to the number of each class living at that age. He showed that the mortality from scirrhus of the breast is 45 per cent. higher in the unmarried than in the married. When we remember that a considerable number who would appear amongst the married are nulliparous, it becomes clear that parous women enjoy a quite important advantage over those who have borne no children in respect of immunity from cancer of the breast. But, further, it is probable that the decisive factor is not child-bearing but suckling, and it is too true that many mothers either cannot or will not suckle their offspring. It is noteworthy that in my own records there are as many as 48 private patients as against 56 hospital cases. In the case of *e.g.* appendicitis the proportion is 1—3. It may be, therefore, that cancer of the breast is commoner in women of the better classes, and it is notorious that whilst probably 95 per cent. of the hospital class suckle their children for the full time, well-to-do mothers frequently do not.

Even in women with cancer of the breast who have borne children and suckled them I have been impressed by the frequency with which one is told that the affected breast had not been used for nursing, for some reason.

In Japan the death rate from cancer of the stomach, and also from cancer of the uterus, is about the same as in this country. But the incidence of scirrhus mammæ is only one-tenth of the British figure. Here 18.6 per cent. of cancer deaths are from disease affecting the breast; in Japan only 1.8 per cent. Why? The readiest reason is that in that country far more women have children, and they usually suckle them for three years.

We cannot explain exactly why non-functioning makes the gland more liable to dangerous changes. It is easy to understand that the flow of milk will wash out any loose cells that may be forming on the walls of the ducts. Perhaps this may have some influence.

Many doctors, and almost the whole of the laity, are firmly convinced that cancer runs in families. The figures quoted by Hoffman after Pearson seem to establish the contrary. Cancer is such a common disease, that it may be mathematically proved that even if there is no such thing as an inherited tendency, we should still see multiple cases in a few families.

*Table showing probability of multiple cancer cases in groups of persons, without any familial tendency.*

| Number of cancer deaths in family. | Per 100 families of six persons (3 m., 3 f.) |
|---|---|
| None. | 47 |
| One. | 38 |
| Two. | 13 |
| Three. | 2 |

Now, a doctor who acts as family physician to say 500 families of average size, and who saw two cancer deaths in each of 65 of them and three others in each of ten families, would probably be firmly convinced that the theory of heredity was true, but he would be wrong.

Put to the same rigid test, the theory of cancer houses must be relegated to the limbo of the unproven. So, too, the theory that cancer is contagious. These facts should be better known. They would relieve the minds of thousands of the relatives of victims of carcinoma.

It has been suggested that repeated injury to the breast by metal supports in corsets may cause cancer, and this is not impossible. But if it were a frequent cause, we should expect the usual site of the growth to be in the lower quadrant.

As a matter of fact, the upper quadrant is most frequently affected.

**The Diagnosis of Scirrhous Carcinoma of the Breast.**—We shall barely mention the signs which everybody knows, and devote our attention particularly to the doubtful and difficult cases.

Unfortunately, any lump in the breast of a woman over 35 is more likely to be malignant than innocent. There are, however, some innocent lumps in the breast which occasionally enable us to rejoice the heart of a patient the day after she has had her operation by telling her good news. These are: cysts, fibro-adenoma, localised mastitis.

But a preliminary question sometimes arises: is there a lump at all? To determine this, the examination must be made with the flat hand and fingers, the latter moving as if playing the piano; we must not pinch up portions of the gland. The "nobbly" breast in which there are no definite lumps as large as a pea, but a universal shotty feeling, may be let off with a caution—a warning that it needs careful watching in the future. Soft wormlike masses beneath the nipple, out of which some secretion can be expressed, are innocent.

A cyst of the breast is much harder than cysts usually are elsewhere. If fluctuation can be got the diagnosis is clear, but doubt often arises. It ought to be operated on. Needling is dangerous. I have seen cancer and cyst associated.

Fibro-adenoma and its varieties (cystadenoma, etc.) is oftener diagnosed than seen. After 35 it is distinctly uncommon. The special feature about it is that it is very mobile within the breast, and can be chased all around with the finger-tip. It has a definite rounded outline.

Localised mastitis forming a definite lump is not so hard as cancer, but can seldom be recognised with confidence.

FIGURE 3.

(Same section, high power. From lower left corner.) Carcinoma spreading along duct, but not yet bursting out. Lumen filled with debris.

FIGURE 4.

(Same section, high power. From lower left corner.) The only typical patch of carcinoma in the breast. Shows cancer cells invading surrounding tissues.

The hall-marks of scirrhus are all dependent on the firmness of the fibrous tissue within it, which has a tendency to contract. Therefore the lump is stony-hard ; the breast may look a trifle smaller than the other, or it may sit a little higher (if the growth is above the nipple). The skin may show a little dimpling. If this is not visible at once, it may be brought out by pushing the skin over the lump, but it is then less reliable. The nipple may show a trifling deviation of its axis towards the lump. At a later stage the nipple is retracted. Many other signs will appear at a late stage, which need not be discussed.

If any of the above hall-mark signs are present, the case definitely is one of scirrhus. Glands in the axilla do not help us in diagnosis, though very important for prognosis. But if none of the above signs are present we must by no means conclude that there is no cancer. There is a large residue of cases in which it is impossible to say with any confidence what the nature of the lump is.

It cannot be too often insisted that absence of pain, or absence of wasting, is of no value whatever in negativing a diagnosis of cancer of the breast. Both are quite late signs.

There are some special cases in which there may be doubt as to what is the matter with the patient. The lump may be very far out, or there may be no lump in the breast at all, but either glands in the axilla, or a bloody discharge from the nipple, or nipple retraction. I have seen a few examples of each of these conditions.

A very far out lump, generally in the direction of the axilla, is usually a cancer if it is stony hard, arising in an aberrant nodule of mammary gland.

If there is a mass of hard glands in the axilla presenting the characters of cancer, but no lump can be found in the breast or elsewhere, the breast is nevertheless probably at fault, and if the glands are still removable the case ought to be treated as mammary carcinoma.

A bloody discharge without any lump is not by itself sufficient evidence on which to base a diagnosis of cancer. In young women it may be let alone; after 35 a local removal is probably safer.

Nipple retraction without a palpable lump is only of importance if it is recent. In that case it is wiser to regard it as evidence of scirrhus, and to operate. If it has been present for years it may be neglected.

We must frankly recognise the fact, therefore, that doubt will fairly often arise as to the innocency or malignancy of a breast case. Under these circumstances, the old rule should guide us, "that any lump in the breast of a woman over 35 is better out than in." Even if it should prove to be innocent, the patient's mind will be relieved; the future development of carcinoma, which in an abnormal breast is a very real danger, will be prevented, and the operation necessary will be a small and safe one.

Every surgeon who is doubtful as to the nature of the swelling, will, of course, cut across it before he proceeds to remove the breast, and in nine cases out of ten the diagnosis becomes quite apparent to the experienced eye. But occasionally it is very difficult to be sure even then. I used, therefore, to have an immediate section cut and reported on, so that one could act at once on the information received. Unfortunately, on more than one occasion the immediate report has been contradicted by the deliberate pathological investigation. It seems better on the whole to act on the naked-eye verdict, and if one is not pretty sure the swelling is innocent to do the complete operation as for cancer. An experienced surgeon need very seldom find that he has done too much if he works on this rule. Anyhow, even that is better than having to operate again a few days later, especially in view of the likelihood that the exploration will have led to the dissemination of more cancer cells, and added to the risks of recurrence.

**Prophylaxis.**—We fear we cannot offer anything better under this heading than to advise women to reduce their risks of getting cancer of the breast by marrying, bearing children, and suckling them for the full period, using both breasts. No doubt this advice is desperately old-fashioned.

Much, however, could be done to reduce the mortality from this disease if the proemial breast was better treated. All such breasts should be carefully watched, and if a definite lump appears, not the lump only but the whole breast tissue should be removed, leaving the skin, pectoral muscles, axillary structures, and probably (for sentimental reasons) the nipple. I have had to treat three patients for scirrhus who had had lumps previously taken out from that breast, but the whole gland not removed. Recently a woman consulted me who had gone incessantly to one doctor after another for so-called interstitial mastitis, always fearing scirrhus, always told over a period of eight years that she need not have anything done, but now there is an advanced cancer.

After one breast has been removed for carcinoma, if the other shows any tendency to develop lumps the whole gland should be removed. In my series, the second breast was affected in three cases. According to Bloodgood,[4] quoting Kilgore, a woman who has had one breast removed stands a 10 per cent. chance of getting cancer in the other, as against a normal woman's 1 to 2 per cent. chance.

**Treatment and Prognosis.**—We shall not stay to discuss details of the operation for cancer of the breast. The procedure is now reduced to a standard. Nor is it worth while tabling a list of the contra-indications to operative treatment, except to say that it is occasionally justifiable to remove the breast, leaving the axilla, even in an advanced case, to save the patient from a local nuisance from pain and foul discharge. Such cases must, however, be picked with discrimination. Of course, they spoil statistics, but that is of small importance.

*Operation mortality.*—Although the modern operation for this complaint looks very formidable, and there is often some shock, the number of cases who die in hospital or in the nursing-home is not large. The figure is usually given as 2 per cent. In my records, out of 106 patients operated on one died; this was on the seventh day, in a very advanced case, in poor general health.

*End-results.*—The published end-results, in unpicked subjects, show a three or five-year "cure" in from 30 to 50 per cent. of the cases operated on.

I have been able to obtain the after-history of 50 out of 56 cases operated on before the end of 1920. Six were lost sight of. Of these 50, 25 are alive and free from recurrence, and 25 are recurred or dead. Of course, three years' immunity does not constitute a cure. One of my patients recurred in the fourth year, and another in the fifth. I saw two or three years ago a woman with a secondary carcinoma in the femur whose breast had been amputated by Mr. Greig Smith; the exact year was unknown, but he died in 1897. No period furnishes an absolute guarantee of safety. But after three years the recurrences are few, and in any case the patient has had good value for the expense and trouble of being operated on if she has three years of freedom from symptoms.

In my series there are:—

5 patients alive and well over eight years.

5 others alive and well over five years.

But it is not fair to the operation to regard it as only saving half the patients in this series. In three cases the breast was only removed for the purpose of getting rid of a distressful local condition, and growth was knowingly left behind. Other cases were very advanced or ultra-malignant.

Some idea can be obtained before operation of the probable risks of recurrence. If the growth has advanced rapidly, and is not very hard, the outlook is grave. The prognosis is much better when no glands can be felt in the axilla. If supra-clavicular glands are felt enlarged, it is doubtful if it is worth while to do anything.

In my series :—

| | No. of cases over three years. | Alive and well over three years. | Dead or recurred. |
|---|---|---|---|
| All cases | 50 | 25 | 25 |
| No glands | 21 | 13 ( =62%) | 8 |
| Glands | 26 | 12 | 14 |

Most of my patients have had a course of X-ray treatment after the operation, but I am unable to adduce conclusive evidence that it makes any difference. Certainly when an obvious recurrence has appeared X-rays do not usually appear to influence it.

Little can be said to guide the practitioner as to how long a patient with carcinoma of the breast, unoperated or recurred, will be likely to live. The observed rate of growth is the principal factor in arriving at a prognosis. The average duration of life from the first appearance is from two to three years, but wide variations are seen from this normal. Atrophic scirrhus in an old woman may last twenty years. On the other hand, in young women, and especially during pregnancy, the growth may go ahead with the most frightful rapidity. Sampson Handley[5] makes the useful observation that when there are signs of involvement of the lung the patients seldom live three months, and never six.

**Encephaloid Carcinoma.**—This condition is interesting, both from the point of view of diagnosis and treatment. It is an ultra-acute form of the disease, and generally the patients are dead in three months. Fortunately, it is not very common.

A doctor who has never seen a case may be congratulated on his astuteness if he makes a correct diagnosis. The whole breast is red, swollen, slightly œdematous, and shows the pitted condition called pig-skin or *peau d'orange*, especially near the nipple. On palpation the breast is not particularly hard. No defined lumps can be felt in it. Axillary glands enlarge at an early date. The unwary will probably make a diagnosis of subacute or acute mastitis, and if there is a little rise of temperature, which may be the case, will probably want to incise it. There is no form of subacute mastitis, however, in which the whole breast is so uniformly involved without any definite and localised abscess-formation.

Three patients with this condition have come under my care. I operated on the first, following the usual technique. It recurred before she left the nursing home, and she was dead in two months.

The next case was several years afterwards. Deterred by the former experience, I advised against operation, and very half-heartedly suggested X-ray treatment. To our astonishment, the benefit was extraordinary. The breast shrunk almost to normal proportions, and the red, angry appearance vanished. We almost dared to hope it might be arrested. But, alas! after many months it became active again; this time X-rays seemed to have no power over it, and she died twelve months after the original consultation.

The third case was seen by all the Infirmary surgeons, who concurred in the diagnosis, and gave a very bad

Figure 5.
Section of encephaloid cancer of breast after X-ray therapy. Many necrotic cells, some unaffected.

Figure 6.
Section from same breast showing many cancer cells beneath the skin, unaffected by the radiation.

prognosis. She was treated by vigorous X-ray therapy, and the growth responded wonderfully. The breast became much smaller and looked less inflamed. Happily, there were no palpable axillary glands. Taking warning by the preceding case, I removed the breast by the usual technique for cancer. The histological appearances made me very glad I had done so. Although the sections showed masses of dead and degenerating cancer cells, there were others very much alive, including some, strange to say, just beneath the skin. The operation was on January 23rd, 1923. To-day, February 23rd, 1924, she is alive, and as far as she knows free from recurrence; that is, eighteen months after the appearance of the first symptoms. This long survival in a case of proved encephaloid carcinoma must be very rare, if not unique.

**Summary.**—1. Like all forms of cancer, this condition in the female breast is increasing. Of women over 35, 7.5 per cent. will get cancer, and in 1.5 per cent. it will affect the breast.

2. Usually the growth arises in the ducts, and follows the condition of hyperplasia called the "proemial breast."

3. It is much commoner in women who have not suckled than in women who have. In Japan it is quite uncommon. Heredity has no influence.

4. The diagnosis depends principally on evidence of contracting fibrous tissue within a lump in the breast, but is often in doubt. Some special cases are considered.

5. Prevention is best served by advising women to marry, bear children, and suckle them properly. If definite lumps appear in the breast the whole breast tissue should be removed subcutaneously.

6. In the series recorded one patient died out of 106 operated on. Of 50 cases followed for three years or more, 25 are alive and well. Of favourable cases 62 per cent. are alive and well. Five patients are alive and well over eight years.

7. In cases of encephaloid carcinoma the best line of treatment appears to be to X-ray first and remove the breast after. One such case is alive and well eighteen months after being first seen (now twenty months).

Figures 1, 2, 3, 4 are from a very early cancer of breast preceded by the condition called " proemial breast."

I am indebted to Dr. A. D. Fraser for the sections, and to Mr. Bolton of the Physiological Department for the photographs.

### REFERENCES.

[1] Hoffman, *The Mortality from Cancer throughout the World.*

[2] Cheatle, *Brit. J. Surg.* 1921, ix. 235 ; *Brit. M. J.*, 1922, i. 869.

[3] Ewing, *Neoplastic Diseases*, 1919.

[4] Bloodgood, " The Diagnosis of Early Breast Tumours," *J. Am.M. Ass.*, 1923, lxxxi. 875–882.

[5] Sampson Handley, article " Breast Cancer," Rendle Short's *Index of Prognosis.*

[6] Keynes, *Brit. J. Surg.* 1923, xi. 89.

### DISCUSSION.

Mr. Carwardine said that he felt himself that the prime cause of carcinoma must be sought in the nucleus of the cell. He pointed out the importance of the penetration of the basement membrane ; this membrane perhaps normally acted as a protection from invasion by epithelial cells. He spoke of the importance of the cystic breast. The cause of carcinoma did not lie wholly in the ducts ; for instance, a case of adenoma with early carcinomatous change. He thought the irritation of stays might be an ætiological factor.

Mr. Firth doubted the value of leaving the nipple when removing the breast for mastitis. He mentioned a case in which a very small cancer was found, and suggested the use of X-rays for chronic interstitial mastitis.

Mr. Adams thought that dimpling of the skin (on attempting to push or pinch it up) over a tumour was not absolutely diagnostic of carcinoma ; it occurred to a certain extent in a normal breast.

In reply, Mr. Short said that he did not profess that every case of carcinoma began in the ducts, but it was the commonest site. Definite dimpling he regarded as pathognomonic of cancer. Although corsets might be a factor in the ætiology, he thought they were not important ; the upper outer quadrant of the breast was much the commonest site for cancer.

---

## Figures and Tables

**Figure 1. f1:**
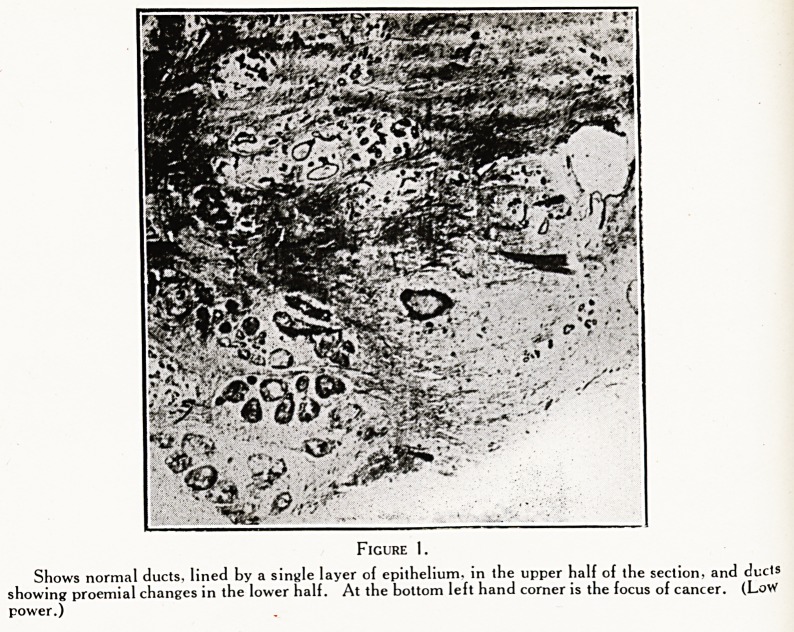


**Figure 2. f2:**
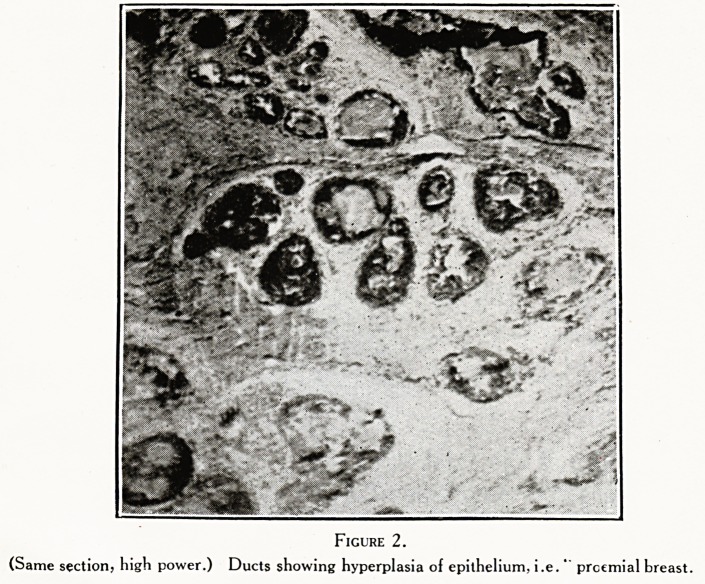


**Figure 3. f3:**
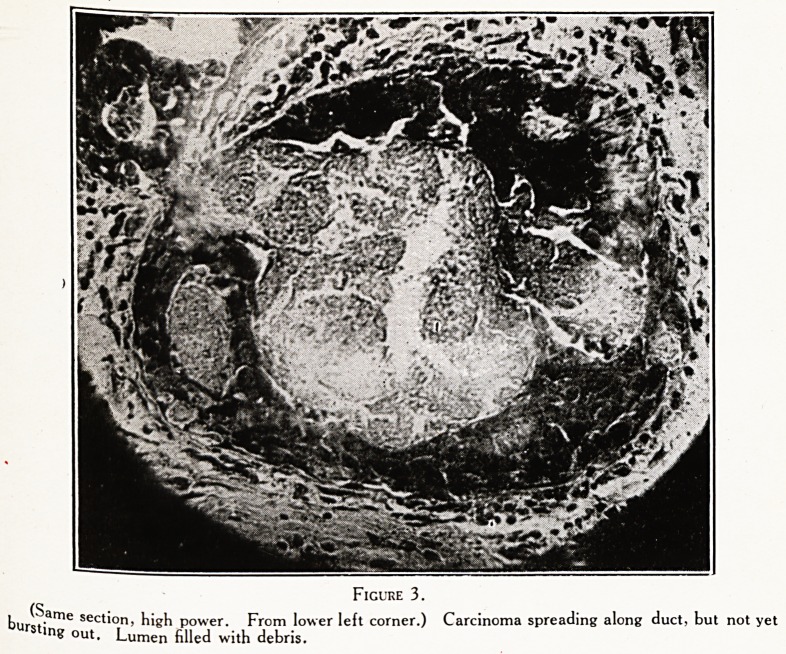


**Figure 4. f4:**
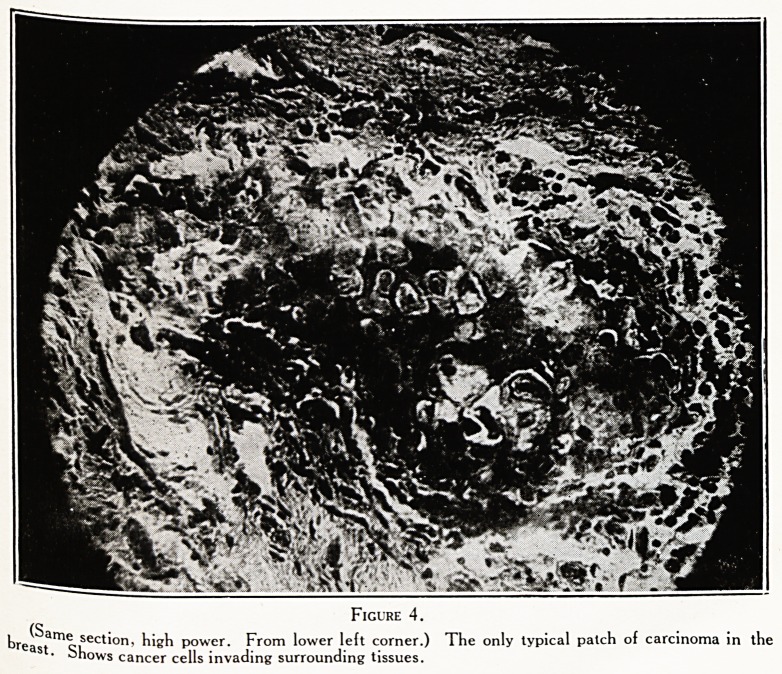


**Figure 5. f5:**
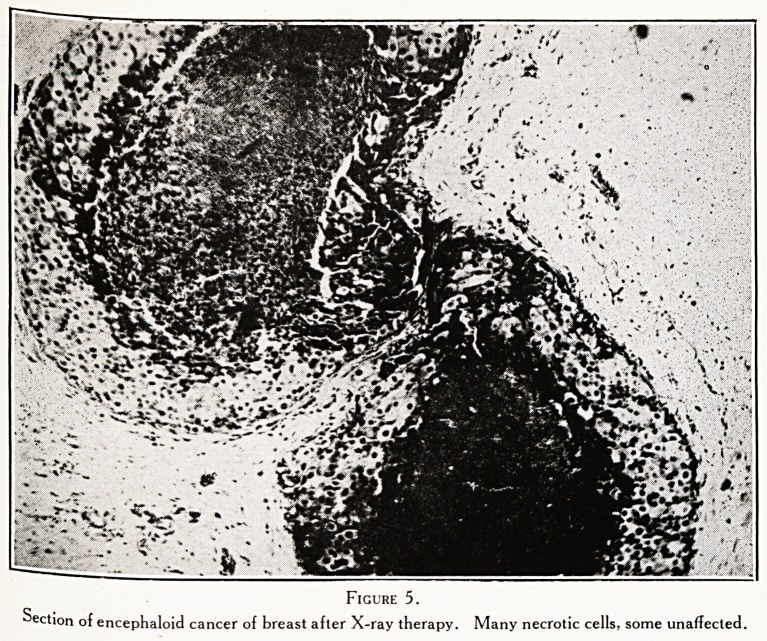


**Figure 6. f6:**